# Effect of novel IL-8 gene mutation on its protein structure and stability among ovarian cancer patients in Saudi Arabia

**DOI:** 10.6026/9732063002001671

**Published:** 2024-11-30

**Authors:** Absarul Haque, Ghazanfar Ali Baig, Abdulelah Saleh Alshawli, Khalid Hussain Wali Sait, Hani S.H Mohammed Ali, Hesham Khalid Hussain Sait, Fehmida Bibi, Peter Natesan Pushparaj, Mahmood Rasool, Muhammad Imran Naseer

**Affiliations:** 1 King Fahd Medical Research Center, King Abdulaziz University, Jeddah, Saudi Arabia; 2 Department of Medical Laboratory Sciences, Faculty of Applied Medical Sciences, King Abdulaziz University, Jeddah, Saudi Arabia; 3 Department of Biological Sciences, Faculty of Science, King Abdulaziz University, Jeddah 21589, Saudi Arabia; 4 Department of Gynecology Oncology Unit, Obstetrics and Gynecology, Faculty of Medicine, King Abdulaziz University Hospital, Jeddah, Saudi Arabia; 5 Special Infectious Agents Unit, King Fahd Medical Research Centre, King Abdulaziz University, Jeddah, Saudi Arabia; 6 Institute of Genomic Medicine Sciences, King Abdulaziz University, Jeddah, Saudi Arabia

**Keywords:** Ovarian cancer, Interleukin-8, Single Nucleotide Polymorphism, automated DNA sequencing, inflammation

## Abstract

Despite advancements in therapeutics, early diagnosis of ovarian cancer (OC) remains a challenge due to its asymptomatic progression and it is often diagnosed at an advanced stage. Interleukin-8 (IL-8), a pro-inflammatory chemokine with angiogenic activity, plays a pivotal role in inflammation and carcinogenesis. Therefore, it is of interest to investigate the association between IL-8 polymorphisms and OC risk. A novel non-synonymous SNP (nsSNP, c.193G>A; p.Glu65Leu) was identified in exon 2 of the IL-8 gene in 5 patients, 3 with stage III and 2 with stage II tumor. Structural analysis suggested that the p.Glu65Leu mutation alters the IL-8 protein conformation showing functional impairment. Thus, data shows that IL-8 c.193 G >A (Glu65Leu) polymorphism may contribute to tumor susceptibility. However, further studies are warranted to understand the role of IL-8 polymorphism in OC.

## Background:

Ovarian cancer (OC) is a leading cause of mortality among gynecological cancers, with five-year survival rates between 40% and 50% [1]. The poor prognosis associated with OC can be attributed to its aggressiveness, diverse histological types and late-stage symptom manifestation [2-3]. The main therapeutic options for OC are chemotherapy, targeted treatments and radical cytoreductive surgery [4]. Histologically, fallopian tubes, ovary and peritoneum are the primary sites for the development of OC [5]. Based on the classification, there are 3 types of OC, including high-grade serous OC (HGSOC), which originates from the tubal epithelium and accounts for 75% of OC cases. Clear cell and endometrioid ovarian carcinoma, also known as endometriosis-related ovarian carcinoma (EROC), arise from endometriotic lesions and provide relatively superior clinical outcomes compared to high-grade serous ovarian carcinoma (HGSOC) [6, 7]. Genetic factors play a substantial role in OC progression, including BRCA1 and BRCA2 alterations, but OC may develop from non-BRCA mutations [8, 9]. Single nucleotide polymorphisms (SNPs) represent prevalent genetic variations, including deletions, substitutions and insertions at specific loci within both coding and non-coding areas. More than 100 SNPs have been observed that significantly contribute to the development of OC [10]. Cytokines and chemokine's act as growth factors in cancer progression through paracrine and autocrine signaling. Their levels typically increase in response to infection or injury and are often expressed by epithelial cells, which are frequent targets of infections [11]. Elevated levels of interleukin-8 (IL-8) and CRP have been detected in ovarian cancer (OC) tissues and ascites, with increased circulating IL-8 levels strongly associated with advanced tumor stages and poor outcomes. High IL-8 concentrations are consistently observed in OC patients [12]. Inflammatory chemokines, especially IL-8 and IL-6, play a significant part in tumor genesis across many malignancies, including OC [13, 14]. IL-8, a member of the CXC chemokine family initially recognized as a neutrophil chemo-attractant, has been shown to play a critical role in tumor growth, invasion, apoptosis and metastasis. It is a key contributor to cancer aggression, angiogenesis and metastatic progression [15, 16, 17-18]. Cancer cells secrete IL-8 in both paracrine and autocrine manners, as observed in ovarian cancer (OC). IL-8's interaction with CXCR1 and CXCR2, two cell-surface G protein-coupled receptors, activates IL-8 signaling pathways [19]. The tumor microenvironment, comprising cancer cells, infiltrating neutrophils, cancer-associated macrophages and endothelial cells, often exhibits elevated IL-8 levels and receptor expression. IL-8 activates angiogenesis in endothelial cells, promoting the survival of both cancerous and endothelial cells while facilitating the recruitment of neutrophils to the tumor site [20]. Elevated IL-8 expression is observed in cancerous ovarian tissues compared to normal tissues. Both OC and stromal cells within the tumor microenvironment are significant sources of IL-8 and concurrently express the CXCR1 and CXCR2 receptors [21, 22]. Eventually, IL-8 has been linked to the progression of chemo resistance through its role in inhibiting apoptosis and fostering the survival of OC cells [23]. IL-8 is located on chromosome 4q12-q135, comprising four exons and three introns and encodes a protein of 99 amino acids. Its 5' promoter region contains multiple nuclear binding sites (NBDs) essential for transcriptional regulation. Nuclear factor κB (NF-κB) regulates IL-8 transcription via TNF receptor-associated factor 6 (TRAF6) and tumor necrosis factor (TNF) [24]. The regulation of IL-8 gene expression and structural variations can significantly impact its functionality. SNPs in the promoter region may alter IL-8 expression, triggering a pro-inflammatory response associated with various tumor phenotypes. Additionally, structural modifications can affect the receptor binding sites, potentially disrupting IL-8's interaction with its targets [20, 24]. Genetic variants in the IL-8 gene influence its expression and protein structure, affecting IL-8-mediated signaling pathways. Notably, Five SNPs have been extensively studied 276A/T, +396G/T, +678T/C, +781C/T and +1633C/T [25]. However, the -251 A > T located in the promoter region and +781 C/T polymorphisms in intron 1, involved in gene regulation and transcription of IL-8, are extensively studied, with implications for IL-8 levels and gene regulation [26]. Therefore, it is of interest to establish the association between IL-8 SNPs and OC risk.

## Materials and Methods:

## Sample collection:

This study was conducted according to a control and case design. Blood samples of control/healthy females were collected. Tissue samples of OC patients were collected from King Abdulaziz University. All the samples were stored at -80°C before use.

## DNA extraction and SNPs analysis of IL-8:

DNA of both OC tissue and blood samples of healthy was isolated using a Genomic DNA Purification Kit (Promega, United States, Cat#A1125) following the manufacturer's protocol. The concentration and purity of DNA were measured using Nano Drop (ND-2000, Thermo Scientific). Isolated DNA samples were amplified using conventional PCR (Applied Bio system Verity) with an exon-specific primer designed and manufactured from Macrogen Inc, South Korea (Forward-5'-AATCCTTAATCACTTTTTCCCCCAA-3', Reverse-5'-ACTACTGTAATCCTAACACCTGGAA-3', annealing temperature 56°C and amplicon size 265 bp). DreamTaq PCR Master Mix (Thermo Scientific, Cat# K1071) was used to amplify DNA. The master mix is a ready-to-use solution containing bacterially derived Taq DNA polymerase, dNTPs, MgCl_2_ and reaction buffers at optimal concentrations to ensure efficient DNA template amplification during PCR. For each run, the thermal cycle conditions were as follows: at the denaturation step, initial denaturation was set at 95°C for 2 min, followed by 35 cycles of denaturation at 95°C for 30 sec, annealing at (50-56°C) for 30 sec, extension at 72°C for 5 min. A final extension was set at 72°C for 5 min and the PCR product was kept at 4°C for an infinite time. Gel electrophoresis was performed to validate the amplification of DNA. SNPs of IL8 were determined by automated DNA sequencing (3500XL genetic analyzer-applied Biosystem). The cycle sequencing PCR reaction was performed using the forward primer according to the manufacturer's protocol (Big Dye Terminator Reaction Kit v3.1, Applied Bio systems). The resulting chromatograms were assessed for quality, with peak analysis compared against reference DNA sequences. SNPs were identified using sequence evaluation software and subsequently confirmed through reverse-strand sequencing.

## Statistical analysis:

The results were statistically analyzed using JASP statistical software v.0.18.3.0 for Windows [34] and the Vassar Stats Web-based tool for Statistical Computation [35]. Fisher's exact test was used to examine differences in genotype frequencies between patient and control groups, while age distribution across study groups was assessed with Student's t-test. A p-value of ≤ 0.05 was considered statistically significant.

## Results:

The mean age of cases and controls was 57.60±14.88 years and 47.30±11.4 years, respectively. High-grade serous carcinoma (HGSC) was the most prevalent histological type, representing 60% of cases. This was followed by low-grade serous carcinoma (LGSC) at 20%, endometrioid carcinoma at 10% and germ cell tumors at 5%. Histological data was unavailable for the remaining 5% of patients. The majority of OC cases (90%) were diagnosed at an advanced stage, with only 5% identified at an early stage. Tumor staging data was missing for 5% of the patients.

## Single nucleotide polymorphism (SNPs):

**Figure 1:**
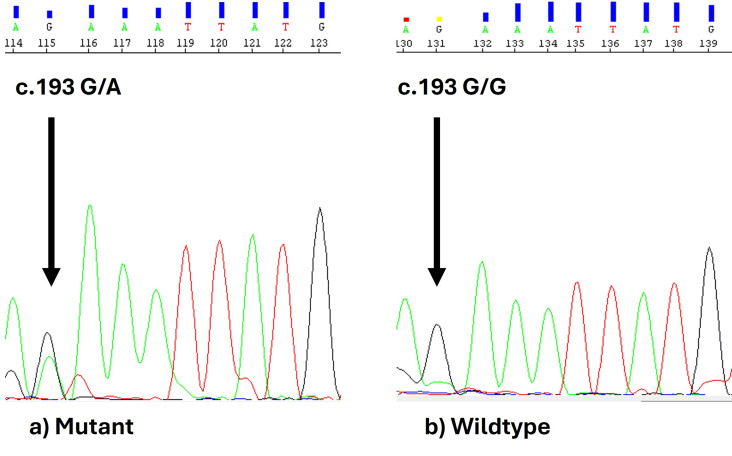
Electropherograms of SNPs identified in Automated DNA sequencing in IL-8 2. a) Mutant c.193G/A and b) Wild type c.193G/G

Genotyping of 20 OC cases and 10 healthy control women was performed using automated DNA sequencing in IL-8 exons 2. We identified a non-synonymous SNP (nsSNP) in exon 2 of the IL-8 gene at position c.193 to 193 G>A, present in 25% of ovarian cancer patients. This nsSNP was found in 5 OC patients diagnosed with advanced-stage OC. The genotyping results are summarized in Table 1. This c.193 G >A nsSNP resulted in the amino acid (AA) change (Glu65Leu). Figure 1 represents the electropherogram of nsSNP identified in automated DNA sequencing.

## Prediction of deleterious nsSNPs and protein stability of IL-8:

We identified deleterious analysis using four SNP prediction algorithms: Polyphen, PROVEAN, PhD-SNP and SNPs & GO. All the web tools predicted that nsSNP (Glu65Leu) may have a deleterious effect, as shown in Table 2.

## Prediction of structural damage using missense 3D:

**Figure 2:**
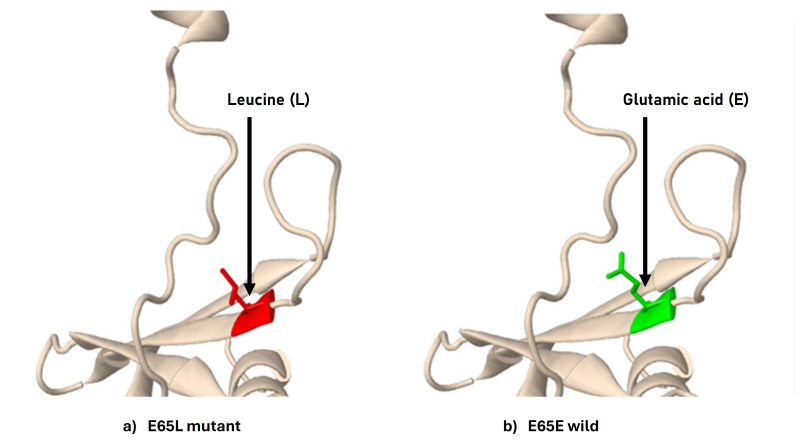
Showing the effect of structural change by the missense mutation, using the missense3D tool. a) Mutant and b) wild type

The damaging impact of mutation Glu65Leu was predicted by the missense3D, as presented in Table 2. In addition, Glu65Leu substitution also predicated the structural damage by buried H-bond breakage by disrupting all side chain/side-chain H-bond(s) and/or side-chain/main-chain bond(s) H-bonds formed by buried Glu residue. The structural change in the wild type and mutant is shown in Figure 2.

## Discussion:

OC is one of the most common causes of death in women due to asymptomatic progression and delay in diagnosis as there is a lack of appropriate diagnostic tools for early detection of tumor [36, 37]. OC is characterized by its strong interaction with the immune system, often initiating a systemic acute inflammatory response pathway [38]. Interleukins are signaling molecules that regulate immune and inflammatory responses. Interleukins play a central role in driving systemic changes, such as inflammation and immune modulation, which contribute to tumor progression and metastasis [39]. Interleukin-8 (IL-8) initially recognized as a neutrophil chemo attractant and a key mediator of leukocyte-driven inflammation, plays a critical role in cancer progression in various cancers including OC [40, 41-42]. Elevated IL-8 levels in serum, ascetic fluid and tumor tissues, along with its overexpression, are strongly associated with poor prognosis and decreased survival in OC patients [43]. Furthermore, IL-8 polymorphisms have been linked to an increased risk of developing OC [44]. Previous studies have primarily focused on two SNPs: -251 A/T in the promoter region and +781 C/T in intron 1, both SNPs play a role in the susceptibility to OC risk [44, 45-46].

However, there is a lack of studies focusing on IL-8 polymorphisms within the coding region of IL-8 in OC. Hence, in this study, we aim to investigate a possible polymorphism in the IL-8 coding region. Therefore, this study aimed to identify potential polymorphisms in the IL-8 coding region and assess their impact. DNA was extracted from tumor tissues of OC patients and blood samples from healthy controls, followed by PCR amplification and automated DNA sequencing. Additionally, bioinformatics analysis was performed to predict the effects of identified SNPs on IL-8 protein structure and stability.

In the current study, we have identified a novel nsSNP at c.193G > A in the IL-8 exons 2, as shown in Figure 1. This mutation involves a nucleotide substitution at position 193 (G to A), leading to an amino acid change from glutamic acid to leucine at position 65, as shown in Table 1. However, statistical analysis showed no significant association between this nsSNP and OC, as shown in Table 1. Hence, studies with larger cohort sizes are warranted to further investigate the correlation between this nsSNP and OC risk. Furthermore, studies have demonstrated significant associations between IL-8 polymorphisms, particularly the +781 (T/T) and +2767 (T/T) genotypes and an increased risk of OC [1, 44]. In addition, *in-silico* analysis was conducted to predict the impact of the amino acid change. Our results indicated that the c.193G>A mutation is likely damaging or deleterious, as determined by bioinformatics tools such as PolyPhen, PhD-SNP, PROVEAN and SNPs & GO, as shown in Table 2. Notably, protein stability analysis using MUPro suggested that the Glu65Leu nsSNP may decrease protein stability. Studies have shown that mutations can lead to protein dysfunction by reducing solubility and impairing function when they cause destabilization beyond a critical ΔΔG threshold [47, 48, 49- 50]. Structural analysis using Missense3D also predicted significant damage caused by the Glu65Leu mutation. This mutation may disrupt essential hydrogen bonds, potentially compromising protein stability.

Thus, our observation revealed that novel nsSNP c.193G>A (Glu65Leu) in the IL-8 exons 2 could have a damaging effect on protein structure/ and function, which may directly impact the interaction with its receptor CXR1\CXR2. Thus, the present in silico functional analysis of this novel deleterious nsSNP c.193G > A, needs to be further validated by correlating the level of IL8 production *in vitro* study before drawing any definite association of this mutation with OC risk.

## Conclusions:

Our findings suggest that IL-8 c.193 G>A (Glu65Leu) polymorphism has potential effect on IL8 structure. However, further investigation of the functional role of IL-8 polymorphism and its impact on the susceptibility of tumor remains ascertained prior to establishing the significance of IL-8 polymorphism as a potential biomarker for OC risk assessment.

## Funding:

The authors extend their appreciation to the Deputyship for Research & Innovation, Ministry of Education in Saudi Arabia, for funding this research work through the project number (1171).

## Informed consent Statement:

Informed consent was obtained from all subjects involved in the study.

## Figures and Tables

**Table 1:** Genotyping analysis of IL-8 polymorphisms in OC tumors and healthy controls

**Gene**	**Exon**	**Samples**	**SNPs**	**Coding sequence**	**Amino acid change**	**Wild Type**	**Nucleotide change**	**Fisher's exact test (p-value)**	**Total**
IL-8 (CXCL8)	2	Cases	c.193G > A	GAA à AAA	p.Glu65Leu	GG (15)	GA* (5)	0.14	15
		Healthy controls				GG (10)	GA (0)		10

**Table 2:** Prediction of nsSNP effects on protein structure and stability using bioinformatics tools

**AA change**	**Polyphen**	**PhD SNP**	**PROVEAN**	**SNPs & GO**	**Polyphen**	**MUPro**	**DDG (Free energy change value)**	**Missense3D**
Glu65Leu	Probably damaging	Disease	Deleterious	Disease	Probably damaging	Decrease	-0.4153387	Structural damage detected (buried H-bond breakage)
